# Supplementary material for: Structural Analysis of Tilvestamab in Complex with AXL
Source: ACS Omega. 2025 Dec 22;11(1):1874–82. doi: 10.1021/acsomega.5c10003 (PMC12809806; doi:10.1021/acsomega.5c10003)
Supplement: Supplementary file 1 [file ao5c10003_si_001.pdf]

Supplementary Information

Structural analysis of tilvestamab in complex with AXL

Eleni Christakou [1,2], Andrea J. Lopez [1], Gopinath Muruganandam [3,4], David Micklem [2], James B. Lorens [1,5], Petri Kursula [1,6,*]

[1] Department of Biomedicine, University of Bergen, Norway

[2] BerGenBio ASA, Bergen, Norway.

[3] VIB-VUB Center for Structural Biology, Vlaams Instituut voor Biotechnologie, Brussels, Belgium

[4] Structural Biology Brussels, Department of Bioengineering Sciences, Vrije Universiteit Brussel (VUB), Brussels, Belgium

[5] Centre for Cancer Biomarkers, Norwegian Centre of Excellence, University of Bergen, Bergen, Norway.

[6] Faculty of Biochemistry and Molecular Medicine, University of Oulu, Finland

*address for correspondence: petri.kursula@uib.no

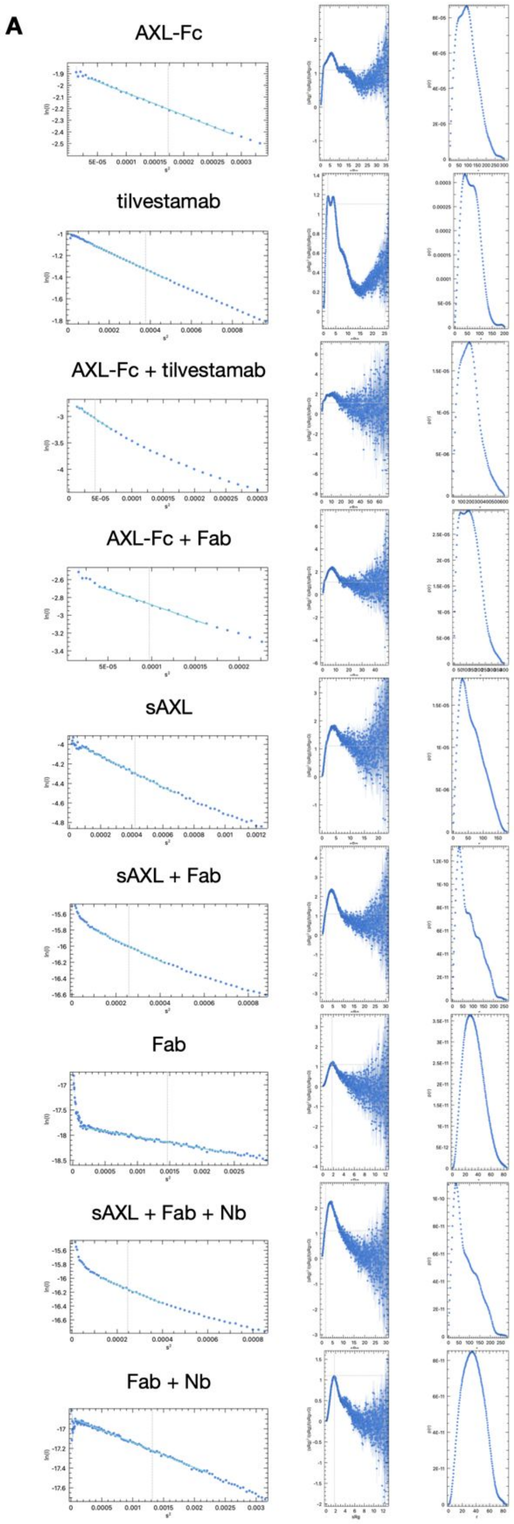

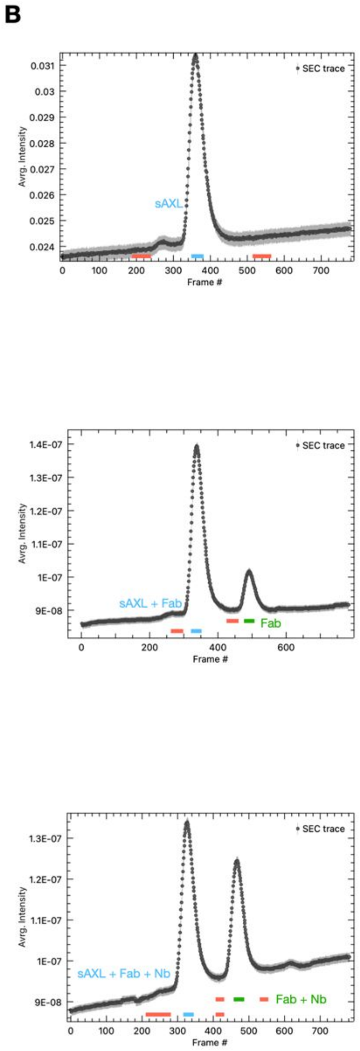

Supplementary Figure 1. SAXS experiment details. A. Guinier plots (left), dimensonless Kratky plots (middle), and distance distributions. **B.** SAXS average intensity traces for the data used for modelling in Fig. 3. Sample frames are indicated in cyan/green and selected buffer frames in red.
